# Accelerated Lactate Dehydrogenase Activity Potentiates Osteoclastogenesis via NFATc1 Signaling

**DOI:** 10.1371/journal.pone.0153886

**Published:** 2016-04-14

**Authors:** Heejin Ahn, Kyunghee Lee, Jin Man Kim, So Hyun Kwon, Seoung Hoon Lee, Soo Young Lee, Daewon Jeong

**Affiliations:** 1 Department of Microbiology, Laboratory of Bone Metabolism and Control, Yeungnam University College of Medicine, Daegu, Korea; 2 Department of Oral Microbiology and Immunology, College of Dentistry, Wonkwang University, Iksan, Korea; 3 Department of Life Science and Research Center for Cellular Homeostasis, Ewha Womans University, Seoul, Korea; Kyungpook National University School of Medicine, REPUBLIC OF KOREA

## Abstract

Osteoclasts seem to be metabolic active during their differentiation and bone-resorptive activation. However, the functional role of lactate dehydrogenase (LDH), a tetrameric enzyme consisting of an A and/or B subunit that catalyzes interconversion of pyruvate to lactate, in RANKL-induced osteoclast differentiation is not known. In this study, RANKL treatment induced gradual gene expression and activation of the LDH A_2_B_2_ isotype during osteoclast differentiation as well as the LDH A_1_B_3_ and B_4_ isotypes during osteoclast maturation after pre-osteoclast formation. Glucose consumption and lactate production in growth media were accelerated during osteoclast differentiation, together with enhanced expression of H^+^-lactate co-transporter and increased extracellular acidification, demonstrating that glycolytic metabolism was stimulated during differentiation. Further, oxygen consumption via mitochondria was stimulated during osteoclast differentiation. On the contrary, depletion of LDH-A or LDH-B subunit suppressed both glycolytic and mitochondrial metabolism, resulting in reduced mature osteoclast formation via decreased osteoclast precursor fusion and down-regulation of the osteoclastogenic critical transcription factor NFATc1 and its target genes. Collectively, our findings suggest that RANKL-induced LDH activation stimulates glycolytic and mitochondrial respiratory metabolism, facilitating mature osteoclast formation via osteoclast precursor fusion and NFATc1 signaling.

## Introduction

Bone consists of a mineral component such as calcium phosphate and other salts as well as an organic component such as collagenous matrix. Bone is a dynamic organ remodeled by a delicate balance between bone-forming osteoblasts and bone-degrading osteoclasts [1]. Osteoclasts, which are multinucleated giant cells formed by cell-cell fusion, contain multiple nuclei (up to 20) and resorb calcified matrix by secreting acids and proteases into the resorption lacuna between the highly convoluted (ruffle border) plasma membrane of the osteoclast and bone surface [2, 3]. Local acidosis in the resorption lacuna dissolves inorganic minerals such as calcium, resulting in the exposure of organic matrix components such as collagen from connective bone tissue [4, 5]. Degradation of the decalcified organic matrix is subsequently carried out by proteolytic enzymes such as collagenases, particularly cathepsin K, and matrix metalloproteinases (MMPs) such as MMP9. Proton transport via ATP input by vacuolar-type H^+^-ATPases (V-ATPase) across the osteoclast ruffle border membrane plays an active role in local acidosis in bone-resorbing areas [6, 7]. Further, osteoclast migration from one resorption site to another is achieved by dynamic rearrangement of the actin and microtubule cytoskeleton, which requires excess ATP hydrolysis [8]. Such high energy demand in osteoclastic resorption indicates that osteoclasts are metabolically active.

Research performed by ourselves and others has found evidence for an active metabolic process in osteoclast differentiation and function as follows: (i) Total cellular RNA and protein contents increase in the receptor activator during nuclear factor-κB ligand (RANKL)-induced osteoclast differentiation, suggesting that differentiation requires a substantial increase in biomass and biosynthetic intermediates to supply cellular constituents [9–11]. (ii) Osteoclastogenic stimulation by RANKL induces a metabolic shift towards accelerated glycolytic metabolism, suggesting that osteoclast precursors undergo elevated glucose influx and lactate efflux, eventually leading to lactic acidosis [9]. (iii) Osteoclasts contain an abundance of mitochondria [12], exhibiting an accelerated tricarboxylic acid (TCA) cycle and mitochondrial respiration to generate more ATP [9]. This is further supported by data showing that metabolic enzymes involved in energy production via the TCA cycle and mitochondrial oxidative phosphorylation are strongly up-regulated during osteoclastogenesis [13, 14]. (iv) Exogenous ATP directly stimulates osteoclast differentiation and resorption pit formation [15], whereas treatment with specific inhibitors (complex I, rotenone; complex III, antimycin A) of mitochondrial complexes that mediate sequential electron transfer or a blocker (oligomycin) for mitochondrial F_0_/F_1_ ATPase suppresses osteoclast formation [9, 16]. These cumulative results suggest that RANKL-induced elevated glycolysis, mitochondrial respiration, and subsequent ATP production are involved in osteoclastogenesis.

Despite some reports that metabolism is essential for regulating osteoclast differentiation and bone-resorbing function, little is known about the role of glycolytic lactate dehydrogenase (LDH) in osteoclast differentiation. Here, we report that up-regulation of LDH activity during osteoclastogenesis promotes both glycolysis and mitochondrial respiration, consequently potentiating mature osteoclast formation via nuclear factor of activated T cell (NFAT) c1 signaling.

## Materials and Methods

### Cell culture and osteoclast differentiation

Bone marrow—derived mononuclear osteoclast precursors were collected from the tibia and femur bones of 6-week-old male C57BL/6J mice (Central Lab Animals, Seoul, Korea) as described previously [17]. Cells were cultured under a humidified atmosphere of 5% CO_2_ at 37°C in bicarbonate-buffered α-MEM (Thermo Scientific, Rockford, IL, USA) supplemented with 10% fetal bovine serum (FBS) and antibiotics. Osteoclast precursors were differentiated into osteoclasts in α-MEM in the presence of M-CSF (30 ng/ml) and RANKL (100 ng/ml) for 4 days with a change of medium after 2 days. On day 2 after osteoclast differentiation, a tartrate-resistant acid phosphatase (TRAP) solution assay to determine extent of pre-osteoclast formation was performed by addition of 5.5 mM *p*-nitrophenyl phosphate, a colorimetric substrate, in the presence of 10 mM sodium tartrate (pH 5.2), and absorbance was measured at 405 nm using a microplate reader. To assess osteoclast differentiation, cells were fixed and stained for TRAP using a leukocyte acid phosphatase staining kit (Sigma-Aldrich, St, Louis, MO, USA) on day 4 after differentiation, and TRAP-positive multinucleated cells (TRAP^+^ MNCs) with more than three nuclei were counted with the use of a light microscope. Further, TRAP^+^ MNCs with ≥ 10 nuclei and ≥ 100 μm in diameter were analyzed as mature osteoclasts. To induce extracellular acidosis, bicarbonate-free α-MEM (Invitrogen, Carlsbad, CA, USA) buffered with 10 mM HEPES and supplemented with 10% FBS and antibiotics was prepared by adjusting the pH to 7.0 or 7.5 with 1 M NaOH. Cells exposed to HEPES-buffered medium were cultured in a humidified atmosphere without CO_2_ at 37°C and further differentiated into osteoclasts in the presence of M-CSF and RNAKL [18]. All animal procedures were approved by the Institutional Review Board of Yeungnam University College of Medicine (YUMC-AEC2014-036) and were performed in accordance with the Guide for the Care and Use of Laboratory Animals. The animals were housed in a room with 22°C–24°C temperature, 60% relative humidity, and maintained on a 24-h light/dark schedule (12:12). All mice were given free access to food and water, and were sacrificed using CO_2_.

### Glucose and lactate assay in culture media

Osteoclast precursors were treated with M-CSF (30 ng/ml) alone or both M-CSF (30 ng/ml) and RANKL (100 ng/ml) for the indicated times. After collecting culture medium, glucose and lactate contents were determined by a colorimetric method using a Glucose Assay Kit or Lactate Assay Kit (BioVision, Milpitas, CA, USA).

### Oxygen consumption (OCR) and extracellular acidification rate (ECAR)

Osteoclast precursors were seeded at a density of 1 × 10^5^ cells/well with α-MEM supplemented with M-CSF (30 ng/ml) in XF cell culture microplates (Seahorse Bioscience, Billerica, MA, USA) and allowed to attach overnight. The next day, cells were incubated in sodium bicarbonate-free α-MEM media (Hyclone, Logan, UT, USA) buffered with 10 mM HEPES (adjusted to pH 7.4 with 1 M NaOH) [18] and supplemented with 10% FBS and antibiotics in the presence of M-CSF (30 ng/ml) alone or both M-CSF (30 ng/ml) and RANKL (100 ng/ml) for 1 h. OCR and ECAR were measured continuously at 37°C using an XF96 analyzer (Seahorse Bioscience), and the readings were collected every 8 min after mixing, waiting, and recording periods in each well. Values obtained were the average of readings for 3 h.

### Quantitative and semi-quantitative RT-PCR

Total RNA was prepared from cells using Trizol reagent (Invitrogen, Carlsbad, CA, USA); 2 μg of total RNA from each sample was reverse-transcribed into cDNA with oligo dT at 42°C for 1 h using a M-MLV reverse transcription kit (Invitrogen) according to the manufacturer’s protocol. Quantitative real-time PCR was performed in triplicate using SYBR Premix Ex Taq (Takara Bio, Shiga, Japan) on an Applied Biosystems 7500 Sequence Detection System and software (Applied Biosystems, San Francisco, CA, USA). Relative mRNA expression levels were determined by the comparative delta threshold cycle method. Expression values of all mRNAs were normalized to the mRNA level of *Gapdh*. Further, semi-quantitative RT-PCR was performed on a Thermo Hybaid PCR Express system (Thermo Hybaid, Ulm, Germany). Primers used are listed in S1 Table.

### Immunoblot analysis and determination of LDH isotypes

Cells were lysed in lysis buffer containing 20 mM Tris-HCl (pH 7.5), 150 mM NaCl, 1% Nonidet P-40, 0.5% sodium deoxycholate, 1 mM EDTA, 0.1% SDS, 1 mM NaF, 2 mM Na_3_VO_4_, 1 mM β-glycerophosphate, and protease inhibitor cocktail (Roche Molecular Biochemicals, Mannheim, Germany). Cell lysates were centrifuged at 10,000 × *g* for 10 min at 4°C, after which protein concentrations of the resulting supernatants were measured by DC protein assay (Bio-Rad, Hercules, CA, USA). Proteins were separated by 10% SDS-polyacrylamide gel electrophoresis, transferred to a nitrocellulose membrane, and probed with specific antibodies. Immune complexes were detected with appropriate horseradish peroxidase—conjugated secondary antibodies and ECL reagents (Abfrontier, Seoul, Korea). Commercially available antibodies were used to detect LDH-A (Abcam, Cambridge, MA, USA), LDH-B (Sigma), Cathepsin K (Abcam), and c-Fos (Cell Signaling, Danvers, MA, USA). Antibodies against p65, NFATc1, DC-STAMP, V-ATPase subunit d2 (Atp6v0d2), and β-actin were purchased from Santa Cruz Biotechnology. To determine LDH isotypes, osteoclast precursors were cultured with M-CSF (30 ng/ml) and RANKL (100 ng/ml) for 4 days. Cytosolic proteins after cell lysis were prepared according to the manual using a Paragon LD gel system (Beckman Coulter, Indianapolis, IN, USA), and 7 μg of proteins was separated by electrophoresis at 50 V for 50 min. The area of LDH isotype activity was visualized using a colorimetric procedure with formazan color reaction.

### Knockdown of LDH by short hairpin RNA (shRNA)

shRNA-mediated knockdown of ARF1 was performed using MISSION Lentiviral Transduction Particles against mouse LDH-A (Sigma-Aldrich, clone ID: TRCN0000041744) and LDH-B (clone ID: TRCN0000041759). Lentiviral transduction was performed based on the manufacturer’s instructions. MISSION pLKO.1-puro control transduction particles (Sigma-Aldrich) were used as control virus particles. After osteoclast precursors were infected with shRNA lentiviral particles or pLKO.1-puro empty control particles in the presence of polybrene (8 μg/ml; Sigma-Aldrich) for 12 h, viral particle-containing medium was exchanged for fresh medium. Infected cells were selected with puromycin (2 mg/ml) for 2 days, and efficient ARF1 knockdown was confirmed using RT-PCR and immunoblot analysis. After successful transduction, cells were induced to differentiate into osteoclasts in α-MEM supplemented with M-CSF (30 ng/ml) and RANKL (100 ng/ml) for the indicated times.

### Fusion Assay

In the cell fusion assay, osteoclast precursors (2 × 10^5^ cells per well) treated with M-CSF (30 ng/mL) and RANKL (100 ng/mL) for 2 days were seeded in 48-well plates to reach 100% confluence and further cultured with M-CSF and RANKL for 3 days. After cells were fixed with 3.7% formalin and stained with TRAP, the number of TRAP^+^ MNCs containing more than three nuclei was counted using a light microscope.

### Statistical analysis

Quantitative data are the mean ± SD from at least three distinct experiments. The data were analyzed by two-tailed Student’s *t* test. For statistical analysis of multiple comparisons, means between groups were performed using one-way ANOVA analysis with the Microsoft 2010 Excel program. A minimal level of *P* < 0.05 was considered to be statistically significant.

## Results

### Glycolysis and mitochondrial respiration are accelerated during osteoclast differentiation

**Fig 1:**
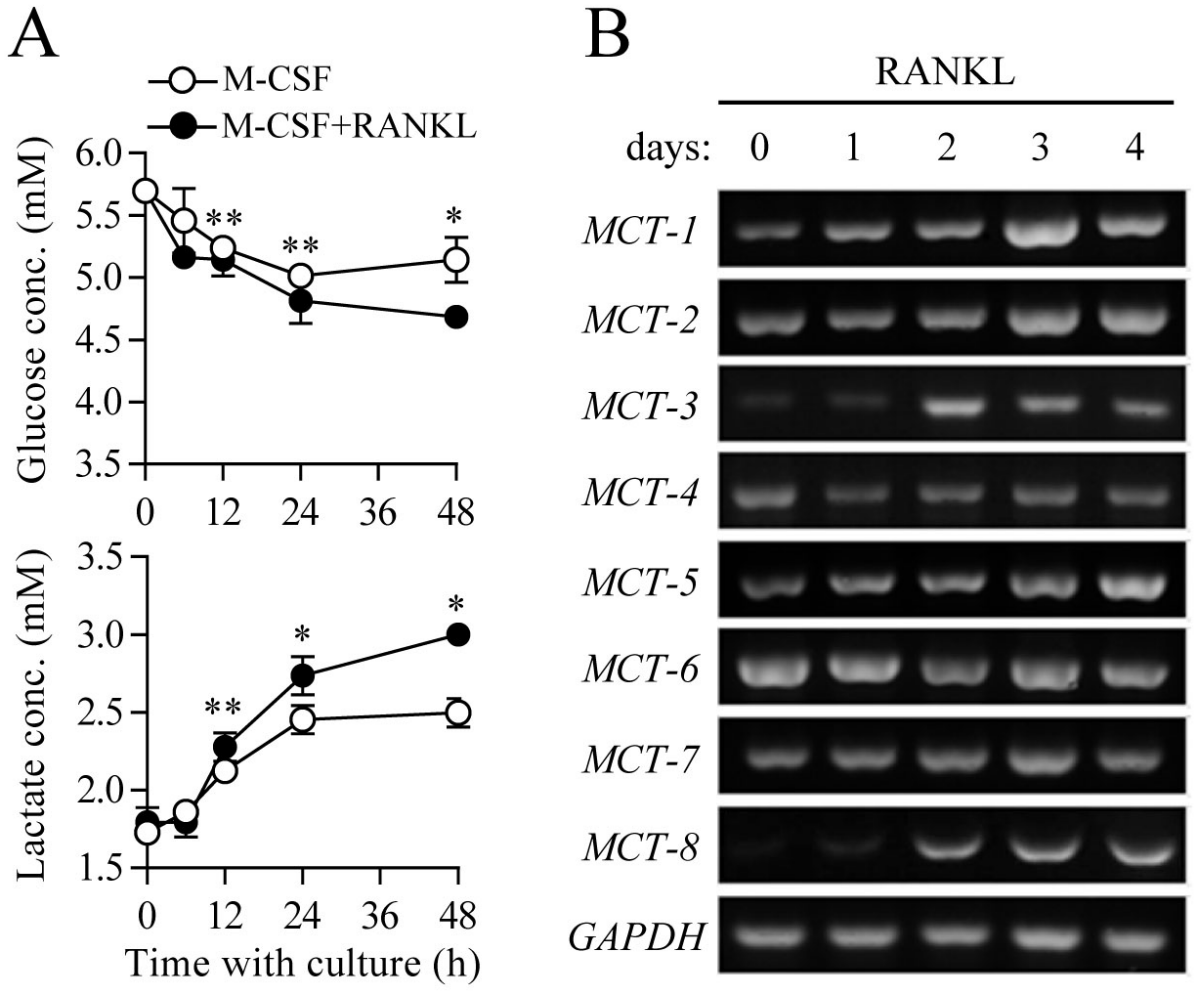
Increased glycolytic metabolism during osteoclast differentiation. Osteoclast precursors were cultured with M-CSF (30 ng/ml) and RANKL (100 ng/ml) for the indicated times. (A) Glucose and lactate contents in cell culture media. Concentrations of glucose and lactate in the culture medium were measured. Data are given as mean ± SD for a representative experiment run in triplicate. *P < 0.01, **P < 0.05. (B) Gene expression analysis of monocarboxylate transporters (MCTs) during osteoclast differentiation. Total RNA isolated from cells was subjected to RT-PCR analysis of the indicated mRNAs. Level of glyceraldehyde 3-phosphate dehydrogenase (GAPDH) served as an internal control for equal loading.

To analyze changes in metabolic activity during RANKL-induced osteoclast differentiation, we first assessed glycolytic activity, as characterized by glucose consumption and lactate accumulation in growth media. RANKL treatment to initiate osteoclast differentiation induced a time-dependent increase in glucose consumption in culture media compared to control (Fig 1A, upper panel). Further, our own report and others showed that among the glucose transporter (GLUT) isoforms present in the cytoplasmic membrane (GLUTs 1 to 4), GLUT1 and 3 are predominantly expressed in the osteoclast precursor stage [9], and GLUT1 is up-regulated in the mature osteoclast stage [10]. These combined results indicate that enhanced influx of glucose from an extracellular compartment into the cytosolic space upon RANKL stimulation may be dependent on the GLUT gene expression level as well as mutual affinity between glucose and GLUT [19]. To identify further metabolic processes involving glucose in cells, we next assessed production of lactic acid, a glycolytic end product that dissociates into lactate anion and a proton (H^+^) at physiological pH and acts as a causative factor in intra- and extra-cellular acidification [20]. RANKL-treated osteoclast precursors showed higher accumulation of lactate in culture media than the control (Fig 1A, lower panel), as evidenced by accelerated extracellular acidification (S1A Fig). It was previously reported that proton-linked monocarboxylate transporters (MCTs) allow the export of lactate anions and protons into the extracellular space to minimize acidic damage in cells [21]. When analyzing gene expression of MCT isotypes (MCT1 to 8), mRNA expression levels of MCT1, 2, 3, 5, and 8 gradually increased during osteoclast differentiation, whereas expression levels of MCT4, 6, and 7 remained unchanged (Fig 1B). Additionally, RANKL-stimulated osteoclast precursors showed marked elevation of oxygen consumption, representing completion of aerobic glucose oxidation via the TCA cycle and electron transport chain reaction in mitochondria, compared to the control (S1B Fig). These results indicate that treatment with the osteoclast key factor RANKL led to a metabolic shift towards elevated glycolysis and mitochondrial respiration, together with increased GLUT and MCT activities mediating glucose influx and lactate efflux, respectively.

### LDH is up-regulated during osteoclast differentiation

**Fig 2:**
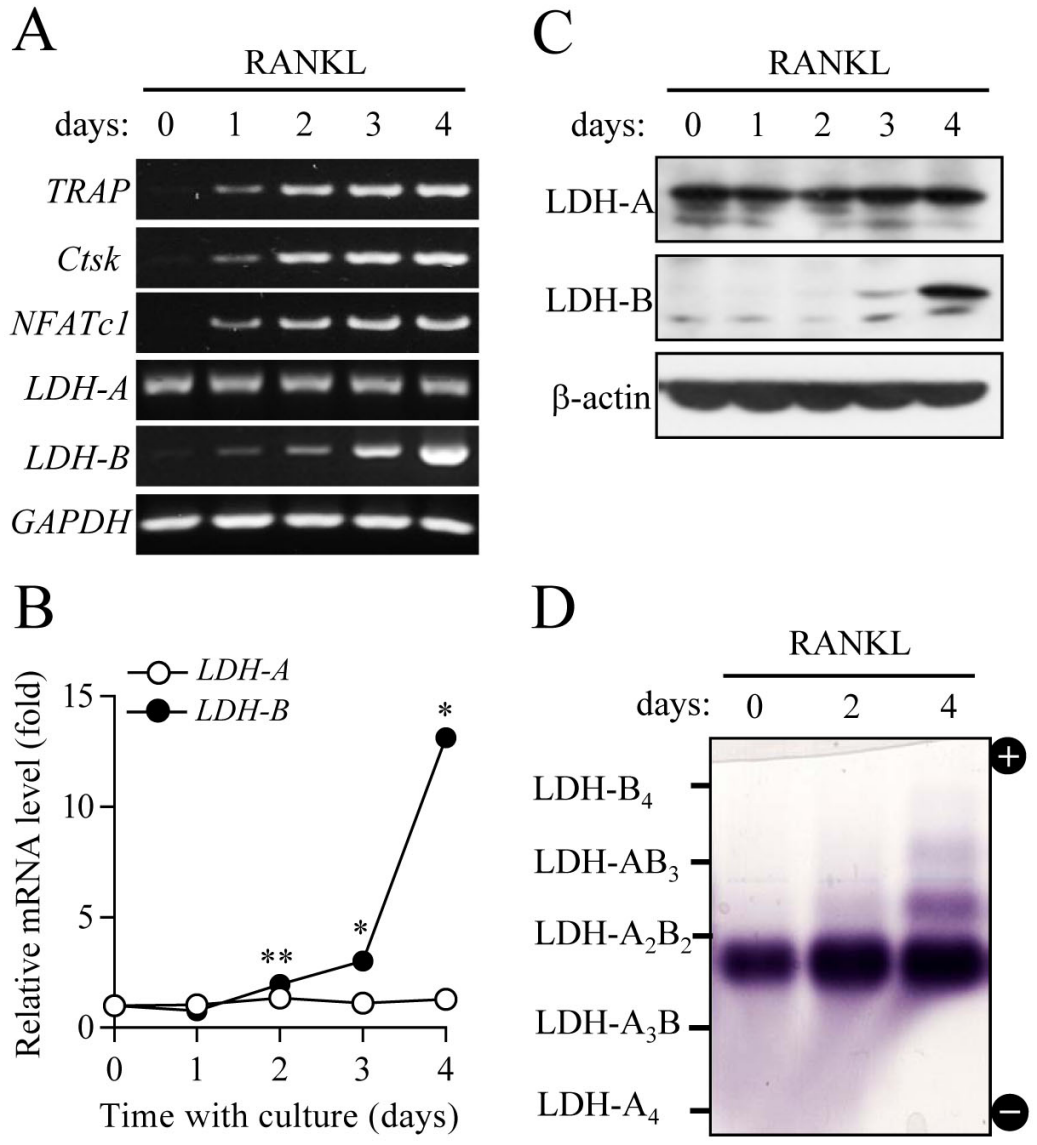
Changes in LDH gene expression and isotypes during osteoclast differentiation. Osteoclast precursors were cultured in the presence of M-CSF (30 ng/ml) and RANKL (100 ng/ml) for 4 days. (A and B) mRNA levels of LDH-A and LDH-B were determined using RT-PCR (A) and quantitative real-time PCR (B). Data for (B) are means ± SD for a representative experiment run in triplicate. *P < 0.01, **P < 0.05. (C) Protein levels of LDH-A and LDH-B were analyzed using immunoblot analysis. GAPDH and β-actin were used as loading controls. (D) Profiling of LDH isotypes. To analyze LDH isotypes, agarose gel electrophoresis was performed, and the activities were visualized by a formazan color reaction.

LDH is a tetrameric enzyme formed by combinations of subunit A and/or B, resulting in five isotypes (LDH-A_4_, A_3_B_1_, A_2_B_2_, A_1_B_3_, and B_4_) that catalyze interconversion of pyruvate to lactate [22]. LDH-A, the predominant form in skeletal muscle, converts pyruvate into lactate, whereas LDH-B is mainly expressed in heart muscle and favors conversion of lactate into pyruvate. The present results that both glycolysis and mitochondrial respiration were elevated during osteoclastogenesis led us to further investigate whether or not LDH is involved in osteoclast differentiation. Along with up-regulation of osteoclast critical genes, including TRAP, cathepsin K, and nuclear factor of activated T-cells cytoplasmic 1 (NFATc1) during RANKL-induced osteoclast differentiation, mRNA expression of LDH-B subunit sequentially increased during differentiation, as determined by semi-quantitative RT-PCR and real-time PCR (Fig 2A and 2B). Likewise, mRNA of LDH-A subunit was equally expressed during differentiation, as further supported by immunoblot analysis of the protein levels of LDH-A and LDH-B subunits (Fig 2C). To analyze changes in the profiles and activities of LDH isotypes, we performed on-gel colorimetric assay using the cytosolic fraction prepared from differential stages of osteoclast differentiation. As shown in Fig 2D, LDH-A_2_B_2_ type was predominantly presented in osteoclast precursors and mature osteoclasts, and its band intensity increased after RANKL treatment, showing elevated LDH-A_2_B_2_ activity. We also observed increased patterns of LDH-A_1_B_3_ and LDH-B_4_ in the mature osteoclast stage. Together, our observations suggest that elevated LDH-A_2_B_2_ activity during osteoclast differentiation can be attributed to increased combinations of LDH-A and LDH-B, whereas augmented combinations of A_1_B_3_ and B_4_ of LDH in mature osteoclasts may be caused by increased gene expression of LDH-B subunit.

### Depletion of LDH-A or LDH-B leads to defective osteoclast formation due to reduced metabolic activity, decreased osteoclast precursor fusion, and NFATc1 down-regulation

**Fig 3:**
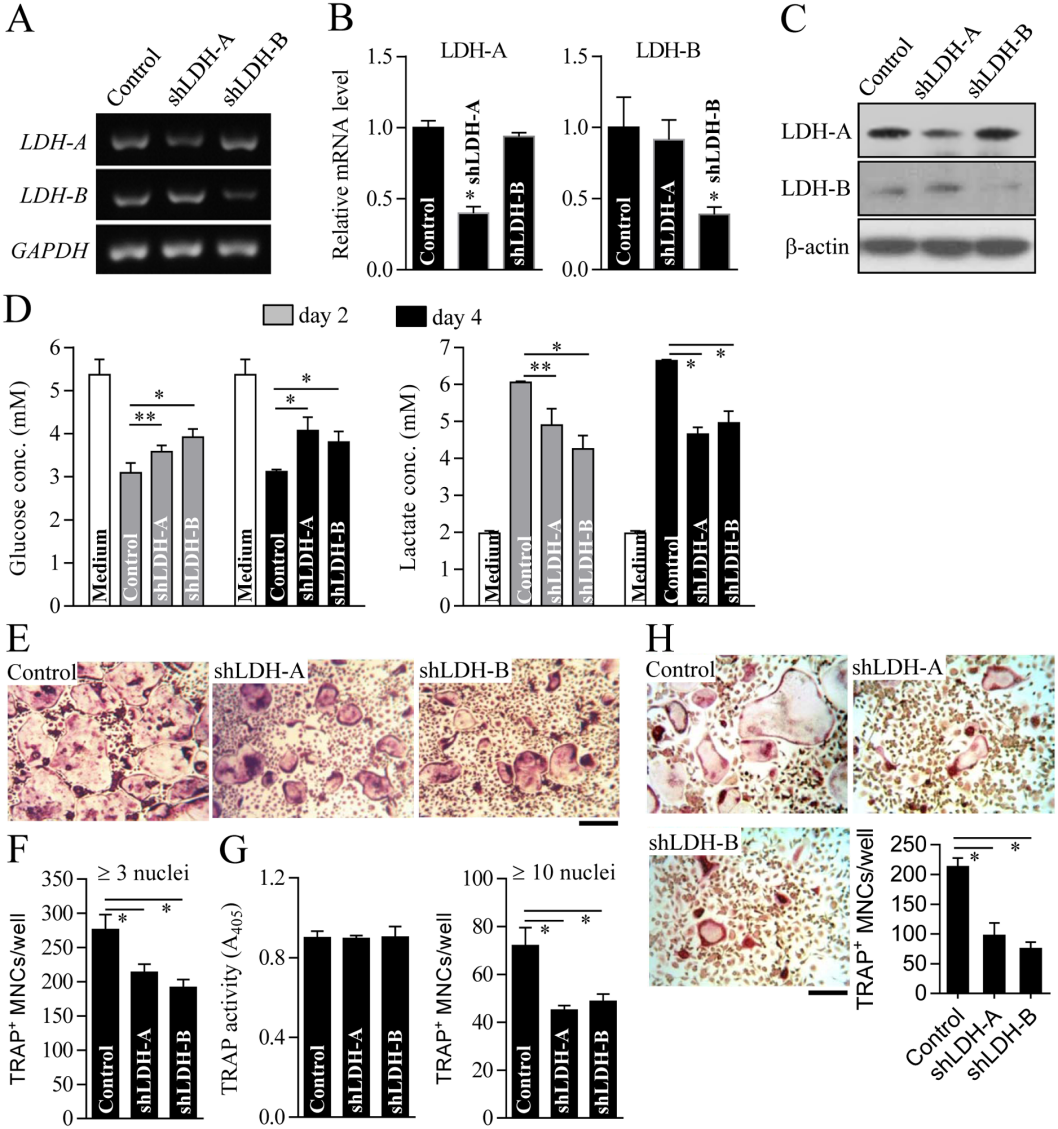
Decreased osteoclast formation by LDH-A or LDH-B depletion. Osteoclast precursors were infected with shRNA lentiviral particles targeting mouse LDH-A, LDH-B, or pLKO.1-puro empty control virus particles. After puromycin selection for 2 days, LDH-A or LDH-B knockdown was validated using RT-PCR (A), quantitative real-time PCR (B), and immunoblot analysis (C). GAPDH and β-actin were used as loading controls. (D) Glucose and lactate concentrations in culture media of LDH-A or LDH-B-depleted cells. LDH-A or LDH-B knockdown cells were cultured with M-CSF (30 ng/ml) and RANKL (100 ng/ml) for 2 or 4 days as indicated. Concentrations of glucose and lactate in the culture media were determined. (E and F) Measurement of osteoclast formation. LDH-A or LDH-B-depleted cells were cultured for 4 days in the presence of M-CSF and RANKL to induce osteoclast formation. Cells were stained with TRAP and photographed using a light microscope (E). Scale bar, 100 μm. The number of TRAP^+^ MNCs with more than three nuclei was counted under a light microscope (F). (G) Determination of TRAP activity and mature osteoclast formation. To assess the extent of pre-osteoclast formation, TRAP activity was measured on day 2 after osteoclast differentiation (left panel). Mature osteoclast formation was determined by counting the number of TRAP^+^ MNCs with more than 10 nuclei (right panel). (H) Cell fusion assay. Osteoclast precursors treated with M-CSF and RANKL for 2 days were seeded and further cultured in the presence of M-CSF and RANKL for 3 days. After TRAP staining, TRAP^+^ MNCs with more than three nuclei were counted using a light microscope. Data are mean ± SD (n = 3). *P < 0.01, **P < 0.05.

**Fig 4:**
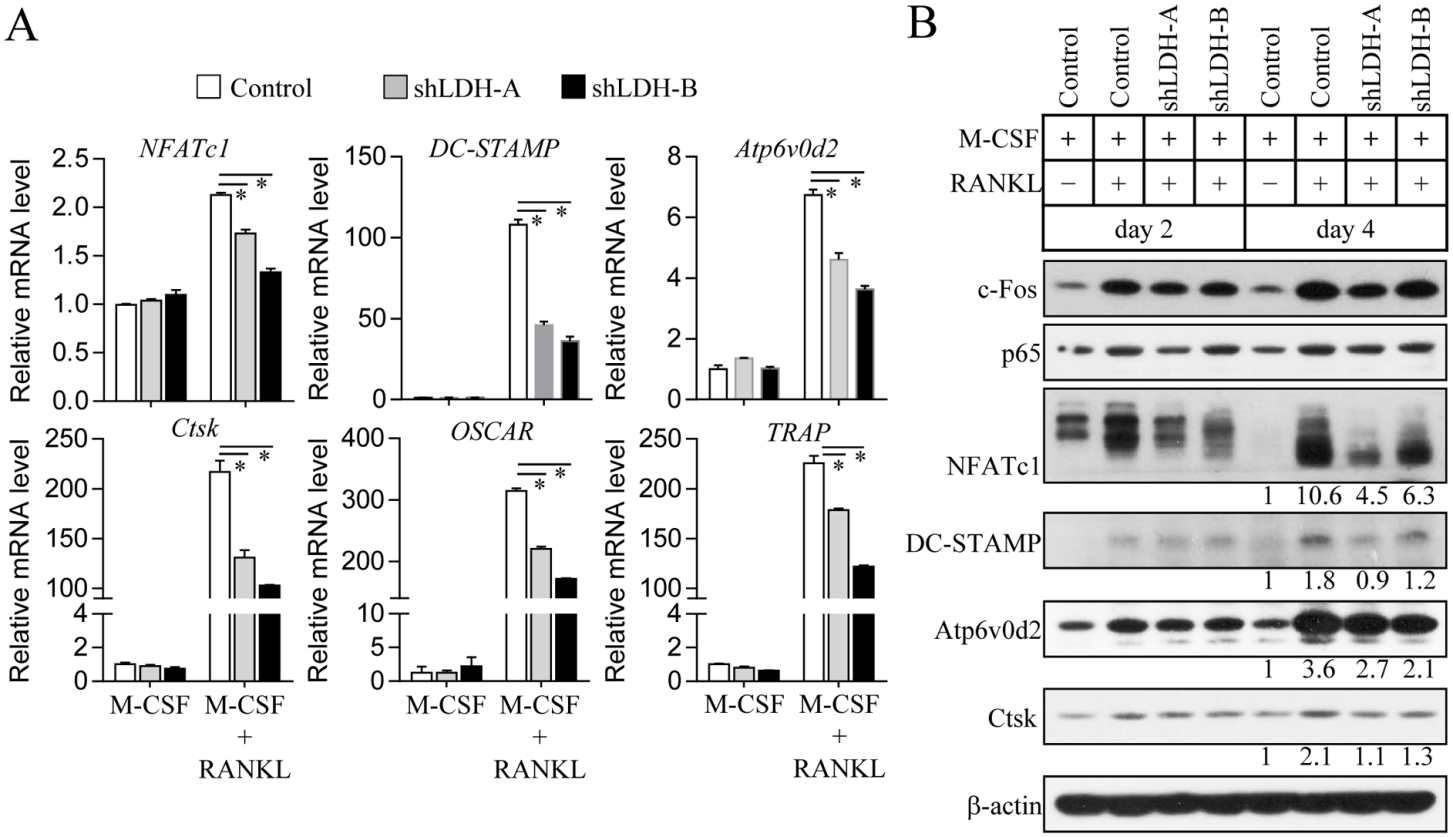
Altered osteoclastogenic signaling during osteoclast differentiation of LDH-A or LDH-B-deficient cells. Osteoclast precursors were infected with LDH-A, LDH-B, or pLKO.1-puro empty control virus particles. LDH-A or LDH-B knockdown cells were cultured with M-CSF (30 ng/ml) and RANKL (100 ng/ml) for 3 days (A) or the indicated days (B). (A) The mRNA expression levels of osteoclastogenic genes including NFATc1, DC-STAMP, Atp6v0d2, cathepsin K (Ctsk), OSCAR, and TRAP were analyzed using quantitative real-time PCR. Data are mean ± SD (n = 3). *P < 0.01. (B) Total cell lysates were subjected to immunoblot analysis with specific antibodies to c-Fos, p65, NFATc1, DC-STAMP, Atp6v0d2, cathepsin K (Ctsk), and β-actin (loading control). Band intensities were represented as a fold difference. Gel images are representative of three independent experiments.

To reveal the functional role of LDH in osteoclast formation, we performed LDH knockdown using lentiviral-delivered shRNAs. Stable knockdown of LDH-A or LDH-B was determined at the mRNA and protein levels, as characterized by RT-PCR (Fig 3A and 3B) and immunoblot analysis (Fig 3C), respectively. LDH-A or LDH-B-deficient cells showed low glucose consumption and lactate accumulation in culture media (Fig 3D), resulting in reduced extracellular acidification (S2A Fig) and decelerated mitochondrial respiration (S2B Fig). Lack of LDH-A or LDH-B resulted in a reduced number of TRAP^+^ multinucleated cells (TRAP^+^ MNCs) with more than three nuclei compared to the control (Fig 3E and 3F). We next assessed whether or not inhibition of osteoclast formation by LDH-A or LDH-B depletion occurs at any stage of osteoclast differentiation. Total TRAP activity on day 2 after osteoclast differentiation, as an indicator of the extent of pre-osteoclast process and formation, was not significantly different regardless of the expression level of LDH-A or LDH-B (Fig 3G, left panel). In comparison, the number of TRAP^+^ multinucleated cells (TRAP^+^ MNCs) with more than 10 nuclei, representing formation of multinucleated mature osteoclasts, was noticeably reduced by LDH-A or LDH-B depletion (Fig 3G, right panel). Osteoclast precursor fusion has been reported to be a crucial step in forming multinucleated mature osteoclasts during osteoclast differentiation [23]. Cell fusion assay of osteoclast precursors showed that depletion of LDH-A or LDH-B resulted in a significant decrease in the formation of TRAP^+^ multinucleated osteoclasts (Fig 3H). These findings clearly indicate that LDH may be substantially involved in mature osteoclast formation. To further explore the molecular mechanism underlying defective osteoclast formation by LDH depletion, we analyzed RANKL-induced osteoclastogenic signaling pathways. As shown in Fig 4, LDH-A or LDH-B-deficient cells treated with RANKL exhibited marked reduction of NFATc1, which is a critical osteoclastogenic transcription factor that mediates osteoclast maturation after the pre-osteoclast stage [24]. The mRNA levels of NFATc1-targeted genes such as TRAP, cathepsin K (Ctsk), OSCAR, V-ATPase subunit d2 (Atp6v0d2), and DC-STAMP were significantly reduced in LDH-A or LDH-B-deficient cells treated with RANKL (Fig 4A). Immunoblot analysis showed that osteoclast-fusion factors DC-STAMP and Atp6v0d2 [25, 26], and bone-matrix proteolytic enzyme cathepsin K (Ctsk) [27] were decreased in LDH-A or LDH-B-deficient cells during osteoclast differentiation (Fig 4B). However, other genes such as the osteoclastogenic transcription factors c-Fos (a component of AP-1) [28] and p65 (a component of NF-κB) [29], showed no significant difference between LDH-depleted cells and the control. Collectively, our results suggest that RANKL-induced LDH activation mediates mature osteoclast formation via the stimulation of osteoclast precursor fusion and the induction of NFATc1 signaling.

**Fig 5:**
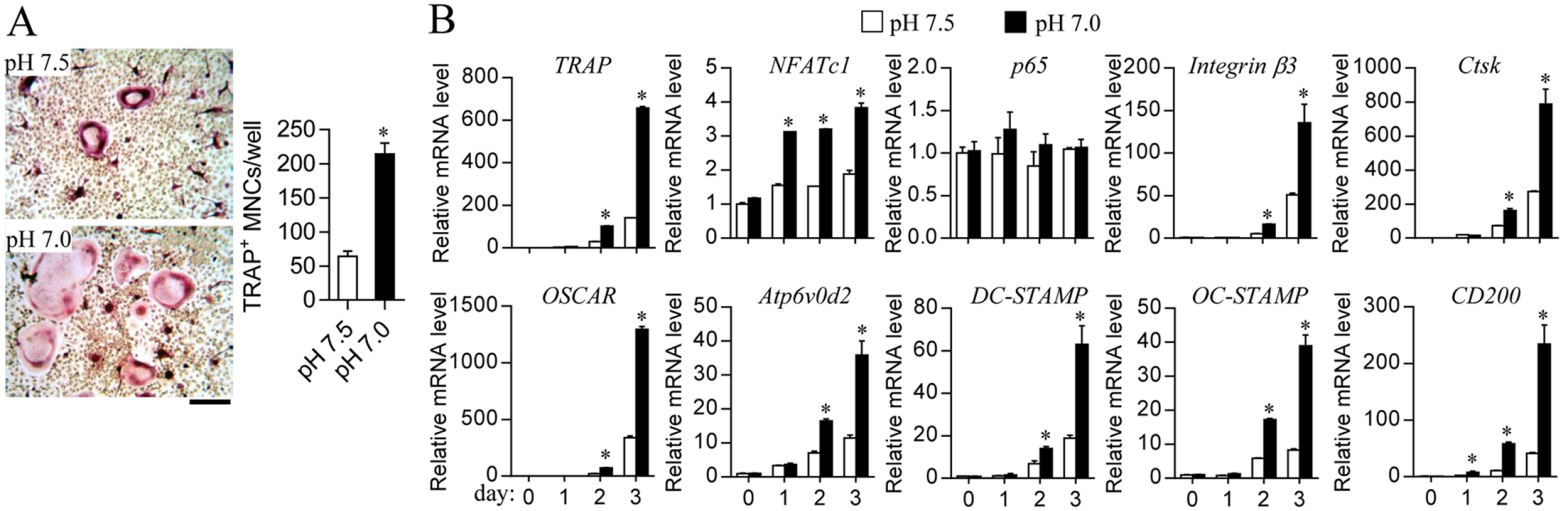
Stimulatory effect of acidic extracellular pH on osteoclast differentiation. Osteoclast precursors were cultured under a CO_2_-free condition in HEPES-buffered medium (pH 7.0 or 7.5) in the presence of M-CSF (30 ng/ml) and RANKL (100 ng/ml) for 4 days (A) or for the indicated times (B). (A) Measurement of osteoclast formation. Cells were stained with TRAP and the number of TRAP^+^ MNCs with more than three nuclei was counted under a light microscope. Scale bar, 100 μm. (B) Analysis of osteoclastogenic gene expression. mRNA levels were analyzed using quantitative real-time PCR.

It has been reported that lactic acidosis due to increased glycolysis leads to acidic extracellular pH, which stimulates osteoclastic bone resorption [18, 30]. To resolve a possible link between LDH and osteoclast differentiation, we finally investigated whether extracellular acidosis resulted from the end product lactic acid of LDH affects osteoclast differentiation. Relatively low pH (pH 7.0) in the culture medium induced an increase in osteoclast differentiation compared to that in pH 7.5 medium (Fig 5A). The osteoclastogenic critical transcription factor NFATc1, target genes of NFATc1 such as TRAP, cathepsin K (Ctsk), and OSCAR, and fusion-related genes including Atp6v0d2, DC-STAMP, OC-STAMP, and CD200 were up-regulated in cells cultured in relatively acidic culture medium (Fig 5B). These results indicate that acidic extracellular pH caused by lactic acidosis may stimulate the expression of NFATc1 and its target genes, and accelerate osteoclastogenesis.

## Discussion

Osteoclast differentiation consists of multiple processes, including initial mononuclear progenitor adhesion and migration along the bone surface followed by fusion to form multinucleated cells. This process requires metabolic reprograming to maintain biosynthetic substrates and energy [9–11]. Bone resorption of polycaryotic mature osteoclasts also requires ATP-coupled proton pumping by V-ATPase into the lacuna area between the osteoclastic ruffle border membrane and bone surface to induce mineral dissolution of old bone tissue [2, 3].

Together with active metabolic re-ordering during osteoclast differentiation, enhanced activity of LDH during osteoclast differentiation was caused by increased gene expression as well as accelerated aerobic glycolysis and mitochondrial respiration. In the cytosolic compartment, increased enzymatic activity of LDH-A subunit, which mediates reduction of pyruvate to lactic acid using NADH, produces abundant glycolytic intermediates to supply cellular constituents and allow ATP generation via aerobic glycolysis to support energy demand [22]. [Note: since you added a citation, changed to present tense since this is a literature fact] As generally reported, mitochondria-rich cells such as epithelial cells show extremely high expression of vacuolar-type proton-pumping ATPase (V-ATPase) both on their plasma membrane and in intracellular vesicles, high expression of cytosolic carbonic anhydrase, and elevated activity of mitochondrial metabolism [12]. Consistently, non-epithelial mitochondria-rich osteoclasts show high levels of V-ATPase on the ruffle border of the cytoplasmic membrane on the bone-resorptive pit region [2, 3], resulting in an acidic microenvironment, as well as increased expression of carbonic anhydrase during osteoclast differentiation [31]. The present study also suggests that enhanced activity of LDH-B subunit, which mediates conversion of lactic acid to pyruvate, induces both biosynthetic substrate synthesis and energy production via the TCA cycle and mitochondrial oxidative phosphorylation. As a result, LDH activation leads to bidirectional induction of glycolysis and mitochondrial respiration in the metabolic adaptation process during osteoclast differentiation.

Although an active metabolic process is critical for osteoclast differentiation and bone-resorption processes, including old bone dissolution by an osteoclastic acidic microenvironment in the lacuna and osteoclastic cell migration to another site, regulation of osteoclast differentiation by specific metabolic enzymes has yet to be elucidated. Here, we showed that increased expression and activity of LDH during osteoclast differentiation induce glycolysis and mitochondrial respiration to facilitate differentiation of osteoclast precursors into mature osteoclasts. Thus, similar to anti-cancer therapeutic agents that can regulate glycolytic enzymes and metabolic intermediates of tumor glycolysis [32, 33], our findings propose that regulators capable of inhibiting metabolically active enzymes (e.g. LDH) can be promising therapeutic targets to treat osteoporotic diseases due to induction of osteoclast differentiation and bone-resorptive function.

## Supporting Information

S1 Table Sequences of PCR primers used in this study. (DOCX)

S1 Fig Measurement of extracellular acidosis and mitochondrial oxygen consumption. After osteoclast precursors were incubated in sodium bicarbonate-free HEPES-buffered α-MEM media with M-CSF (30 ng/ml) and RANKL (100 ng/ml) for 1 h, extracellular acidification rate (ECAR) (A) and oxygen consumption rate (OCR) (B) were measured continuously at 37°C using a XF96 analyzer. ECAR and OCR readings were collected every 8 min, and values are the average of readings for 3 h. Data are presented as mean ± SD (n = 3). *P < 0.01, **P < 0.05.
(TIF)

S2 Fig Changes in ECAR and OCR by LDH-A or LDH-B depletion. Osteoclast precursors were infected with shRNA lentiviral particles targeting mouse LDH-A, LDH-B, or pLKO.1-puro empty control virus particles and then selected with puromycin for 2 days. Cells were treated as in S1 Fig and ECAR (A) and OCR (B) were measured. Data are presented as mean ± SD (n = 3). *P < 0.01, **P < 0.05.
(TIF)
