# Supplementary material for: Accelerated Lactate Dehydrogenase Activity Potentiates Osteoclastogenesis via NFATc1 Signaling
Source: PLoS One. 2016 Apr 14;11(4):e0153886. doi: 10.1371/journal.pone.0153886 (PMC4831772; doi:10.1371/journal.pone.0153886)
Supplement: S1 Table — (DOCX) [file pone.0153886.s001.docx]

**S1 Table. Sequences of PCR primers used in this study.**

| Semiquantitative RT-PCR | | | | |
| --- | --- | --- | --- | --- |
| Gene | Sense (5′→3′) | | Antisense (5′→3′) | |
| *MCT-1*  *MCT-2*  *MCT-3*  *MCT-4*  *MCT-5*  *MCT-6*  *MCT-7*  *MCT-8*  *LDH-A*  *LDH-B*  *TRAP*  *Ctsk*  *NFATc1*  *GAPDH* | TGGGGGTGGGCAGTGTTAGTC  TCTGTGCGTCCTTTATCTCCATTG  CATCTTCTACCTTGCTGGCT  AGGCGGTCAGCGTCTTTTTCAA  AGGATGGGGGTGGATGGTC  ACTGCTGGCCCGATATCTTCTGGA  ACAAAAGGCGTTCGGTAGTCA  CTGGATAGTGGTGTTTGCAGC  GGGGCCCGTCAGCAAGAG  GTGGGCGTTGGACAAGTGGGTATG  ACTTCCCCAGCCCTTACTACC  CTTGTGGACTGTGTGACT  TGCTCCTCCTCCTGCTGCTC  CAAGGCTGTGGGCAAGGTCA | | TTTAGGAGGAGGCCCCCAAGAAT  CTCCCCTTCCAGCCATAGTTGTTA  CACTGTGAACAGGGTCTAAC  TGGGGTCCGCGGGGCTCAGT  AGAACGGGCAATTGCTGTAGAAA  ATTGGTGAGCCCGTTGATGAGGAT  GCGGGGCTTTCATCTGG  GGCGACGTTGAAAGTAGTGG  CCAATGGCCCAGGATGTGTAACC  TGAGGTAGCGGAATCGAGCAGAAT  TCAGCACATAGCCCACACCG  AACACTGCATGGTTCACA  CGTCTTCCACCTCCACGTCG  AGGTGGAAGAGTGGGAGTTGCTG | |
| Quantitative RT-PCR | | | | |
| Gene | | Sense (5′→3′) | | Antisense (5′→3′) |
| *LDH-A*  *LDH-B*  *ATP6v0d2*  *DC-STAMP*  *β3*  *NFATc1*  *p65*  *TRAP*  *Ctsk*  *OSCAR*  *OC-STAMP*  *CD200*  *GAPDH* | | GAGGTTCACAAGCAGGTGGT  TGGCATTCATCCCAGCAG  TCAGATCTCTTCAAGGCTGTGCTG  TGGAAGTTCACTTGAAACTACGTG  GATGACATCGAGCAGGTGAAAGAG  GAGACAGACATCGGGAGGAAGA  GCGTACACATTCTGGGGAGT  CTGGAGTGCACGATGCCAGCGACA  ACGGAGGCATTGACTCTGAAGATG  GCTGGTAACGGATCAGCTCCCCAGA  CCTTGGTGCTACAGGCCTAC  AGCCCAGAAAACATGGTCAC  AGGTCGGTGTGAACGGATTTG | | TTTCCACCATGATTAAGGGTC  TGCCTATGAAGTCATCAAGCT  GTGCCAAATGAGTTCAGAGTGATG  CTCGGTTTCCCGTCAGCCTCTCTC  CCGGTCATGGATAGTGATGAGTAG  GTGGGATGTGAACTCGGAAGA  GTTAATGCTCCTGCGAAAGC  TCCGTGCTCGGCGATGGACCAGA  GGAAGCACCAACGAGAGGAGAAAT  CCAAGGAGCCAGAACCTTCGAAACT  CAGAGTCCGAGTTCCCTGTC  AAGGCAAGCTGTTCCTGAGA  TGTAGACCATGTAGTTGAGGT |
