# Supplementary figures and images for: Accelerated Lactate Dehydrogenase Activity Potentiates Osteoclastogenesis via NFATc1 Signaling
Source: PLoS One. 2016 Apr 14;11(4):e0153886. doi: 10.1371/journal.pone.0153886 (PMC4831772; doi:10.1371/journal.pone.0153886)

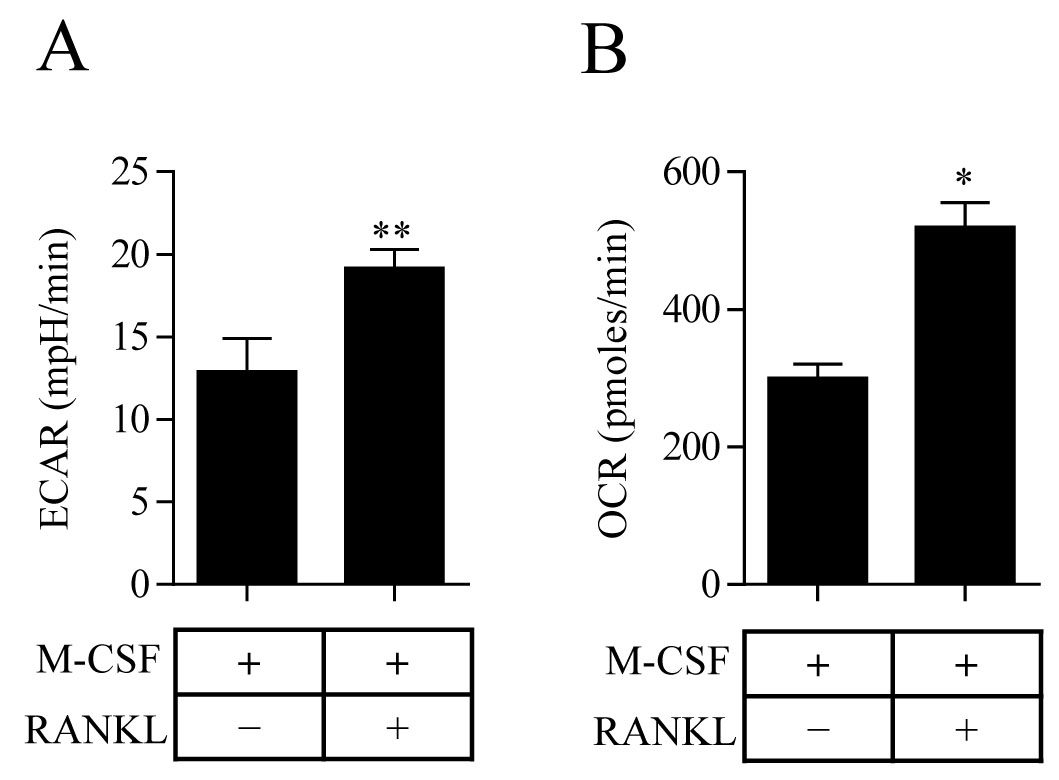

Supplement: S1 Fig — After osteoclast precursors were incubated in sodium bicarbonate-free HEPES-buffered α-MEM media with M-CSF (30 ng/ml) and RANKL (100 ng/ml) for 1 h, extracellular acidification rate (ECAR) (A) and oxygen consumption rate (OCR) (B) were measured continuously at 37°C using a XF96 analyzer. ECAR and OCR readings were collected every 8 min, and values are the average of readings for 3 h. Data are presented as mean ± SD (n = 3). *P < 0.01, **P < 0.05. (TIF) [file pone.0153886.s002.tif]

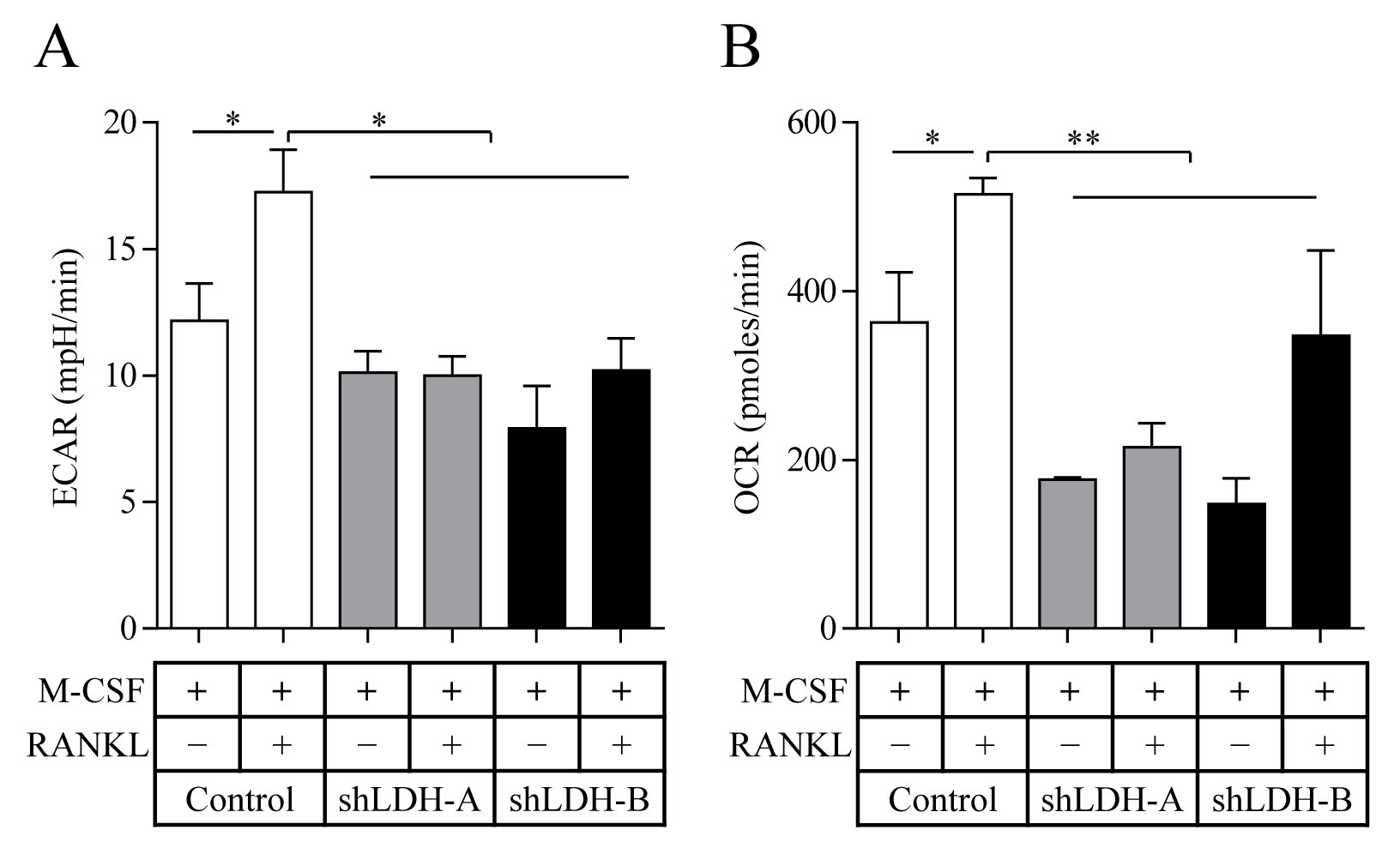

Supplement: S2 Fig — Osteoclast precursors were infected with shRNA lentiviral particles targeting mouse LDH-A, LDH-B, or pLKO.1-puro empty control virus particles and then selected with puromycin for 2 days. Cells were treated as in S1 Fig and ECAR (A) and OCR (B) were measured. Data are presented as mean ± SD (n = 3). *P < 0.01, **P < 0.05. (TIF) [file pone.0153886.s003.tif]
